# The International Medical Annual and Practitioners’ Index

**Published:** 1900-09

**Authors:** 


THE INTERNATIONAL MEDICAL ANNUAL AND PRACTITIONERS' INDEX.—A Work of Reference for Medical Practitioners. 1900. New York: E. B. Treat & Co., 241 West 23d Street. 748 pages. Price, $3.00.

These annuals have been running for eighteen years and have become so well known that the profession, in large part, depend upon them for reliable reviews. There are forty contributors who make up the work, covering the whole range of medicine and surgery. The volume compares favorably with any of the preceding.

